# Insecticidal activity of *Bacillus thuringiensis* towards *Agrotis exclamationis* larvae–A widespread and underestimated pest of the Palearctic zone

**DOI:** 10.1371/journal.pone.0283077

**Published:** 2023-03-16

**Authors:** Jakub Baranek, Magdalena Jakubowska, Elżbieta Gabała

**Affiliations:** 1 Faculty of Biology, Department of Microbiology, Adam Mickiewicz University in Poznań, Poznań, Poland; 2 Department of Monitoring and Signalling of Agrophages, Institute of Plant Protection-National Research Institute, Poznań, Poland; 3 Institute of Plant Protection-National Research Institute, Poznań, Poland; Benemérita Universidad Autónoma de Puebla: Benemerita Universidad Autonoma de Puebla, MEXICO

## Abstract

*acillus thuringiensis* is an entomopathogenic bacterium commonly used as a bioinsecticide against numerous invertebrate pests. However, the efficacy of this microbe has not yet been determined towards *Agrotis exclamationis*–a lepidopteran, polyphagous pest, widespread throughout the Palearctic zone. In this work we have detected very low susceptibility of *A*. *exclamationis* to *B*. *thuringiensis* commercial strains, used as microbial formulations in pest control. To investigate this matter, the biological activity of six selected (Cry1Aa, Cry1Ca, Cry1Ia, Cry2Ab, Cry9Ea and Vip3Aa), heterogously-expressed *Bacillus thuringiensis* insecticidal proteins has been assessed towards *A*. *exclamationis*. Only Cry9Ea and Vip3Aa caused significant mortality in the tested pest species, with LC_50_ values of 950 and 140 ng/cm^2^, respectively. The histopathological effects of Cry9Ea and Vip3Aa on *A*. *exclamationis* were determined. On the other hand, Cry1- and Cry2-type toxins, which are the main active molecules of the majority of currently-used *B*. *thuringiensis*-based biocontrol agents (including the commercial strains tested in this work), did not cause mortality in target insect, but only different levels of growth inhibition. Moreover, in the case of Cry1Ca and Cry1Ia hormesis has been observed–a phenomenon that may be disadvantageous in implementation of these proteins in pest management. The obtained results broaden the existing knowledge regarding *B*. *thuringiensis* insecticidal protein target range and depict variable susceptibility of *A*. *exclamationis* to different groups of Cry/Vip toxins. This work indicates Cry9Ea and Vip3Aa as good candidates for efficient biological control of *A*. *exclamationis* and possibly other *Agrotinae* and discusses the potential use of Vip3-type and Cry9-type insecticidal proteins as successful bioinsecticides.

## Introduction

*Agrotis exclamationis* L. (Lepidoptera: Noctuidae) known as the heart-and-dart moth is a lepidopteran insect species, widespread throughout the entire Palearctic region except Iceland [1–8]. *Agrotis exclamationis* is a herbivore and its voracious larvae (cutworms) are able to graze through leaves, buds and stems, often destroying the entire plants [9]. Like many other members of the Noctuidae family, *A*. *exclamationis* is a serious pest feeding on various economically important crops such as beet, cereals, mustard, potatoes, spinach [7], carrot, swede, strawberry, turnip [9], lettuce, loganberry [10], ornamental trees and shrubs [11] as well as seedling trees in forest nurseries and garden flowers [10]. *Agrotis exclamationis* causes significant losses every year [12], and very often occurs together with cutworm larvae of other closely related species *i*.*e*., *Agrotis segetum* [5, 6, 11, 13]. For example, in Poland the cutworm caterpillars can damage between 2 and 30% of the yield, depending on the crop [14–16].

Currently, to combat insect pests (including lepidopteran larvae such as *Agrotis* cutworms) large amounts of sprayable synthetic pesticides are used all over the world [17]. However, decades of scientific investigations have proven that the use of synthetic chemicals is extremely dangerous to the environment [18] and human health [19]. Worldwide recognition of these threats has led to significant changes in pest control, leading to introduction of Integrated Pest Management concept–an increasing global trend aiming, among others, in reducing the use of synthetic pesticides and replacing them with other control measures, such as bioinsecticides [20, 21]. This gave an opportunity for biopesticide market to grow [22, 23].

One of the best alternatives to synthetic pesticides are bioinsecticides based on insecticidal toxins produced by *Bacillus thuringiensis* (*Bt*). This is a rod-shaped bacterium commonly known for its entomopathogenic properties [24]. The biocidal activity of this species towards invertebrates is attributed mainly to Cry and Vip protein toxins [25]. To date, hundreds of distinct Cry and Vip proteins are known and are classified upon their amino acid sequence similarity to groups and subgroups. Each Cry or Vip protein is assigned a specific four-rank name (*i*.*e*., Cry9Ea11) [26]. *Bt* Cry and Vip toxins bind specifically to certain receptors in insect midgut, and feature relatively narrow spectrum of target organisms. As an example, Cry1, Cry2, Cry9 and Vip3-type proteins are toxic to lepidopterans [27], however certain Cry/Vip protein often targets only a portion of species within the given order. Although significant part of these proteins have been tested for their insecticidal activity, they were mostly assessed against very limited number of pest species [28, 29]. To date, susceptibility of many economically-important insect herbivores to *Bt* proteins has not been studied at all. Such is the case of *A*. *exclamationis*, where, despite the well-documented abundance of this pest in Eurasia and northern part of Africa and substantial crop losses caused by this organism, its susceptibility to *Bt* insecticidal proteins has not yet been determined. Thus, no *Bt*-based, nor other bioinsecticides (*i*.*e*., viral or fungal formulations, biochemical pesticides, etc.) have been developed to combat *A*. *exclamationis*, so far.

Typically, *Bt*-based bioinsecticides can be used in two forms: microbial formulations and transgenic plants producing *Bt* toxins in their tissues (the *Bt*-crops). Endospores and Cry toxin-rich parasporal crystals, derived from different *Bt* subspecies, are active ingredients of the majority of the formulated sprayable microbial insecticides available commercially [23]. In the first decade of 21^st^ century more than 400 of these products were registered worldwide [30]. Biopreparations designed to combat lepidopterans are usually based on *Bt* subspecies such as *kurstaki* (active ingredient of agents such as Foray, Biobit, Dipel, Javelin), *aizawai* (active ingredient of agents such as Selectgyn, Tobaggi, XenTari) or *thuringiensis* (active ingredient of Bacillex, Bathurin, Insectin, etc), as referenced earlier [31]. Another biocontrol option is the use of *Bt*-crops. Since the appearance of the first commercially available transgenic event producing Cry toxin, certain *Bt* insecticidal protein genes were introduced into numerous economically-important crops such as corn, cotton, potatoes, and rice [31]. Soon afterwards the adoption rate of many *Bt* crops was reaching 100% [23] and the area planted with these transgenic events exceeded 1 billion acres worldwide [32]. Till 2014 most of the grown cultivars produced *Bt* Cry1-type toxins (mainly Cry1A, Cry1C and Cry1F) as well as Cry2A-type proteins [33].

Despite the wide variety of *Bt*-based bioinsecticides, they are often ineffective against certain pests, due to the very high specificity of *Bt* insecticidal toxins, among other possible reasons. Even closely related species (*i*.*e*., representing the same genus) can show different level of sensitivity to a given *Bt* protein. For example, Cry1Ca, which is highly active against *Spodoptera exigua*, has been proven ineffective against *S*. *frugiperda* [34]. Therefore, the results cannot be extrapolated between different species and bioassays are always required to determine the exact specificity and insecticidal potency of a given *Bt* Cry/Vip protein. Another pressing matter, regarding *Bt*-based bioinsecticides, are the growing reports of insect resistance development to widely used *Bt* toxins. Especially, the transgenic *Bt*-crops are recognized as exerting high selective pressure and causing resistant insect populations emergence [35]. The main reason for the high rate of insect resistance is the relatively low diversity of Cry toxins (usually Cry1-type or Cry2-type, as referenced above) used in *Bt*-crops. Judging on the above, it is necessary to extend the knowledge regarding various *Bt* insecticidal proteins with low mutual sequence similarity and differences in mode of action in order to successfully implement them in crop protection. This strategy can overcome insect resistance and sustain the use of *Bt*-derived bioinsecticides [36].

The aim of this work was to assess the biocidal potential of *Bt* towards larvae of *A*. *exclamationis*–a lepidopteran crop pest, never tested for its susceptibility to this entomopathogenic bacterium before. First, we determined the insect mortality and growth inhibition caused by two Bt strains (HD-1 and HD-2, representing *Bt* subspecies *kurstaki* and *thuringiensis*, respectively), used for the production of commercial bioinsecticides, as shown above. Next, the insecticidal activity of six selected, heterogously expressed *Bt* insecticidal toxins (Cry1Aa, Cry1Ca, Cry1Ia, Cry2Ab, Cry9Ea and Vip3Aa) has been assessed. The chosen proteins represent four main *Bt* protein groups (namely Cry1, Cry2, Cry9 and Vip3), which are considered lepidopteran-active [27], cluster the highest number of holotype toxins [37] and were most broadly studied [28, 38], so far. Upon dose-response trials the LC_50_ value was determined for each toxin. Moreover, growth inhibition caused by the tested proteins on *A*. *exclamationis* larvae was measured. Finally, the histopathological effects of the most active proteins on insect midgut have been visualized. The results of this work extend the existing knowledge regarding *Bt* toxin host range and upon the gathered information, certain toxins can be selected for application in biocontrol strategies against *A*. *exclamationis*, including Integrated Pest Management.

## Materials and methods

### Insects

*Agrotis exclamationis* imago forms were obtained using light traps from natural environment in Winna Góra (52°12′ N, 17°27′ E), Średzkie County, Municipality of Środa Wielkopolska, Poland. Traps composed of an AC powered glow tube (250 WMI mix), were set up throughout the pest presence in natural environment (May-October). The moths were caught during the night from 9 p.m. to 6 a.m. The light traps were controlled three times a week and the moths (males and females) picked from the traps were systematically segregated and marked.

For mating and oviposition the moths were kept in 5-liter glass containers with cotton-fiber-cloth cover allowing air circulation. The containers were filled with perennial ryegrass (*Lolium perenne* L.) and filter paper, on which the eggs are abundantly laid and which enables their proper collection. The adult insects were fed with 10% sucrose solution. The eggs laid on filter paper were collected every day and kept for hatching in Petri dishes. The hatched larvae were transferred to 900 mL plastic boxes and fed fresh sugar beet leaves before they were used in bioassays. Insects (imago forms and larvae) were reared in phytotron room under controlled conditions (temperature: 21 ±2°C; relative air humidity: 50–70%; photoperiod of 18 h light and 6 h dark). Light of ~53 000 lux was provided using SON-T Agro 400 W lamp.

### Insecticidal protein production

*Bacillus thuringiensis* (*Bt*) subspecies *kurstaki* HD-1 and *Bt* subspecies *thuringiensis* HD-2 were kindly provided by Bacillus Genetic Stock Centre (BGSC), Columbus, Ohio. HD-1 strain (BGSC 4D1) produces Cry1Aa, Cry1Ab, Cry1Ac, and Cry2Aa toxins [39] and the HD-2 strain (BGSC 4A3) harbors genes encoding different set of lepidopteran-active toxins: *cry1Ab*, *cry1Ba*, (GenBank RefSeq assembly accession: GCF_002911755.1). Spore-crystal mixtures derived from the above-mentioned strains were obtained as described in S1 File. Microscopic observation confirmed production of endospores and parasporal crystal bodies in both isolates (S2 File).

For heterologous expression of Cry and Vip insecticidal proteins the pLATE11 vectors were used (Thermo Scientific), containing one of the genes encoding: Cry1Aa (GenBank MK391629); Cry1Ca (GenBank MK391630); Cry1Ia (GenBank MK391631); Cry2Ab (GenBank MK391632); Cry9Ea (GenBank QBO24622) or Vip3Aa (GenBank QKE59889.1) toxin. The construction of the vectors was described earlier [40]. The proteins were expressed in *E*. *coli* BL21 (DE3) codon+ cells and subsequently extracted from lysates according to procedures detailed in S1 File. The expected molecular weights of the proteins have been confirmed via SDS-PAGE (S2 File).

### Insect bioassays

Bioassays were performed on the first instar (2-4-day-old) *A*. *exclamationis* larvae. Insects in the first instar developmental stage has been chosen, because it was assumed that their susceptibility to insecticidal toxins will be higher than in later stages (*i*.*e*., L2, L3, etc.) and thus any toxic effects will be more evident. Bioassays were done by diet surface contamination, using 12-well plates containing lepidopteran-dedicated diet [41]. Diet in each well was covered with 30 μl of a given toxin solution, and allowed to dry. Subsequently, one larva was put per well and the plates were kept in phytotron room with environmental conditions as described above. Three concentrations of *Bt* HD-1 and HD-2 spore-crystal suspensions (850 000, 8500, 850 ng/cm^2^) and five concentrations of Cry/Vip toxins (5000, 1000, 100, 10 and 1 ng/cm^2^) were tested. To each tested concentration 12 (in case of Cry1Aa, Cry1Ca, Cry1Ia, Cry2Ab) or 16 (in case of Cry9Ea and Vip3Aa) larvae were assigned and the bioassays were done in three independent repetitions. Bioassays with spore-crystal suspensions were not repeated, however 24 larvae were assigned per one toxin concentration instead. Demineralized MilliQ water was used as negative control, when testing spore-crystal mixtures. Cleared lysate from non-transformed *E*. *coli* BL21 (DE3) codon plus cells (obtained as described in S1 File) was used as negative control in bioassays performed with heterogously-expressed Cry/Vip toxins. The mortality was scored seven days after treatment (DAT) and an insect was considered dead, when no movement was seen after slight agitation with a brush. There was no mortality in the negative control associated with spore-crystal mixture treatments, whereas the mean mortality in the negative controls associated with Cry/Vip toxin treatments reached only 4.4%. Therefore, the correction of the mortality in treated larvae was omitted. The 50% lethal concentration (LC_50_) values of Cry/Vip protoxins were calculated upon insect mortality in BioStat ver. 6.9.1.0 (AnalystSoft) and they were considered significantly different when the fiducial limits did not overlap. To determine larval growth inhibition caused by Cry/Vip proteins, the larvae surviving 5000, 1000 and 100 ng/cm^2^ treatments as well as the control insects from two independent repetitions were weighed in 0.1 mg precision balance. The growth inhibition of larvae surviving spore-crystal mixtures derived from strains HD-1 and HD-2 (at concentrations of 850 000, 8500, 850 ng/cm^2^) was measured similarly. The mean body weight of toxin-treated larvae was expressed as percent of control larvae body weight. To determine significance of body weight differences between toxin treated larvae and the control, a t-test was used at α = 0.05 level. As positive controls, the spore-crystal mixtures of *Bt* strains (at a concentration of 50 000 ng/cm^2^) and insecticidal toxins (at a concentration of 1000 ng/cm^2^) were bioassayed against susceptible insect larvae (namely *Cydia pomonella* or *Spodoptera exigua*), according to procedures described previously [42, 43]. All strains and toxins were found active (S3 File).

### Histopathological effects of insecticidal proteins on larval midgut

Second instar larvae were fed with artificial diet containing 1000 ng/cm^2^ of either Cry9Ea or Vip3Aa toxin. Simultaneously, the negative control was performed using non-transformed *E*. *coli* cell lysate instead of *Bt* toxin preparations. After 24 h (±4) of incubation (environmental conditions as described above) larvae were subjected to microscopic examination. For ultrastructural analysis in transmission electron microscopy the control and treated larvae were anaesthetized with CO_2_ and immediately fixed in 2.5% glutaraldehyde in 0.1 M phosphate buffer, pH 7.2, at room temperature for 4 h. Next, insect midguts were dissected and rinsed several times in 0.1 M phosphate buffer and post-fixed with 1% osmium tetroxide in the same buffer for 2 h at room temperature. Subsequently, the midguts were washed in water, dehydrated in a graded acetone solution series, and finally embedded in Epon resin. Ultrathin sections were examined with the Hitachi HT7700 (Hitachi, Tokyo, Japan) at 80 kV.

## Results

### Insect bioassays

The activity of two *Bt* strains (the HD-1 and HD-2) against *A*. *exclamationis* larvae was limited (Table 1). Even at the highest concentration, the tested strains did not cause more than 50% mortality, despite inducing substantial reduction of larval growth. However, two lower concentrations of *Bt* spore-crystal mixtures administered to larvae were not larvicidal at all, and caused only minimal reduction of *A*. *exclamationis* development.

**Table 1:** Insecticidal activity of spore-crystal mixtures derived from *Bacillus thuringiensis* subspecies *kurstaki* HD-1 and *Bacillus thuringiensis* subspecies *thuringiensis* HD-2 towards first instar larvae of *Agrotis exclamationis*, scored seven days after treatment.

Strain	Concentration^a^	Mortality (%)	Weight^b^ (%)
Negative control	0	0	-
HD-1	850 000	41.7	18.1
	8500	0.0	95.1
	850	0.0	77.9
HD-2	850 000	20.8	17.1
	8500	0.0	83.5
	850	0.0	79.6

^a^concentration of spore-crystal mixture derived from *Bacillus thuringiensis* strain expressed in ng/cm^2^.

^b^mean body weight of surviving larvae relative to untreated control.

Six distinct, heterologously-expressed *Bt* Cry/Vip proteins exerted different degrees of insecticidal activity against *A*. *exclamationis* larvae (Table 2). The results show that Vip3Aa protein is the most toxic towards *A*. *exclamationis*, with LC_50_ value almost 7-fold lower than the LC_50_ value estimated for Cry9Ea, the next most potent *Bt* insecticidal protein tested. The remaining *Bt* proteins Cry1Aa, Cry1Ca, Cry1Ia and Cry2Ab administered at the highest tested concentration caused >50% mortality.

**Table 2:** Insecticidal activity of *Bacillus thuringiensis* proteins towards first instar larvae of *Agrotis exclamationis*, based upon mortality scored seven days after treatment.

Toxin	a ± SE^a^	b ± SE^b^	LC_50_ (FL min–max)^c^
Cry1Aa	-	-	>5000
Cry1Ca	-	-	>5000
Cry1Ia	-	-	>5000
Cry2Ab	-	-	>5000
Cry9Ea	1.0 ± 0.1	2 ± 0.1	950 (597–1596)
Vip3Aa	0.7 ± 0.1	3.5 ± 0.1	140 (78–257)

^a^Slope of the regression line with standard error.

^b^Intercept of the regression line with standard error.

^c^Lethal concentration causing death of 50% of the treated larvae (LC_50_) with 95% fiducial limits (FL) is expressed in ng/cm^2^.

**Fig 1:**
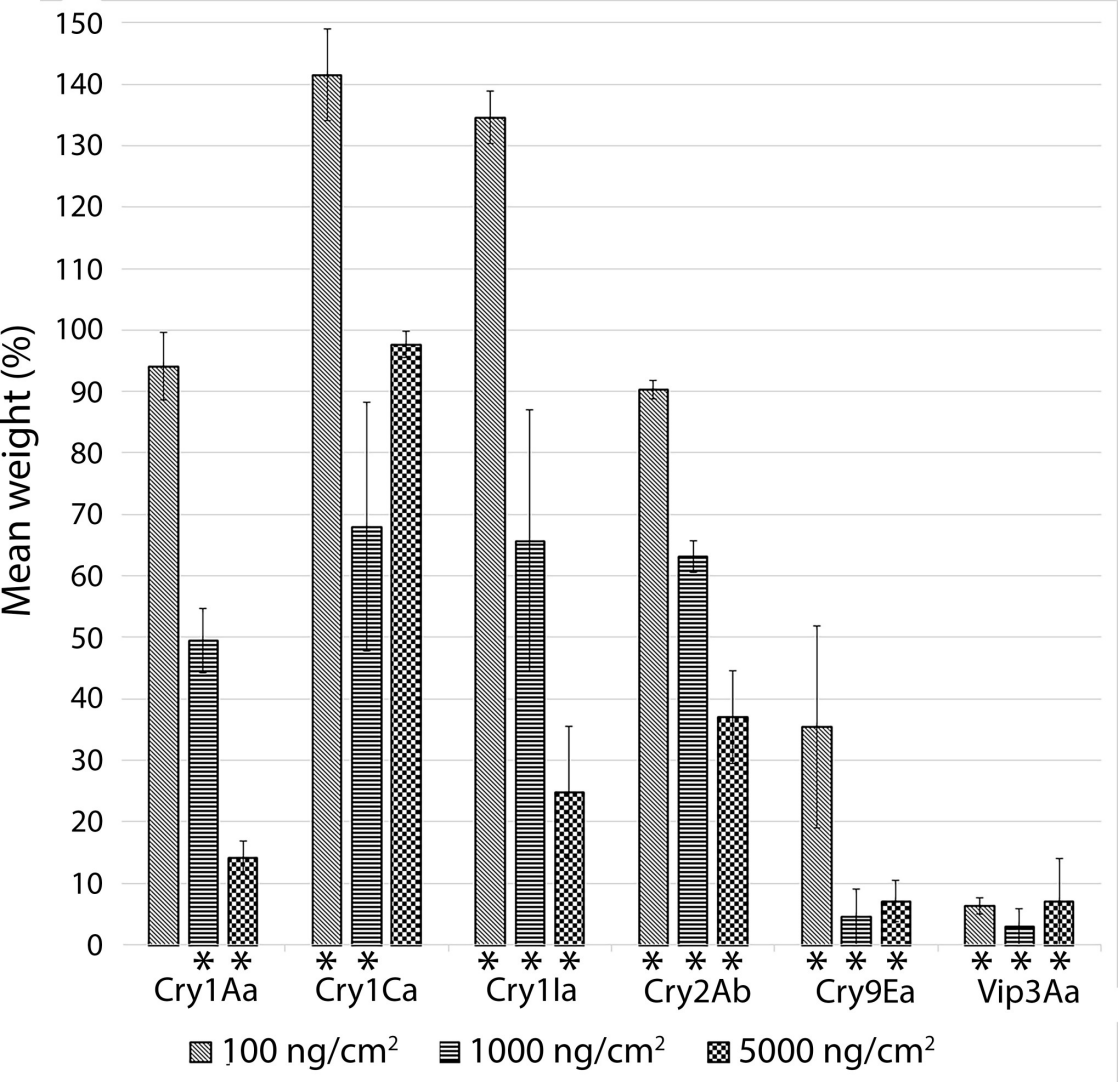
Weight of *Agrotis exclamationis* surviving larvae exposed to *Bacillus thuringiensis* Cry and Vip protoxins in relation to control. Bars and whiskers indicate means ± SD of two replicate trials. Three concentrations used for each protein are indicated in the figure legend. Significant differences in weight between treated larvae and the control, based on t-test (p<*0*.*05*), are indicated with asterisks.

Along with the mortality measurement, also the non-lethal effect of *Bt* toxins on *A*. *exclamationis* larvae was assessed. Comparison of the weight of surviving larvae between treatments and the control showed high variability in larval response to different Cry/Vip toxins (Fig 1). The Cry9Ea and Vip3Aa proteins caused the highest growth inhibition. However, also the Cry1Aa, Cry1Ia and Cry2Ab caused significant larvae development impairment when administered in certain concentrations (Fig 1). In the case of Cry1Ca protein, a significant larval development impairment was noted only for 1000 ng/cm^2^ concentration. Noteworthy are the results obtained for Cry1Ca and Cry1Ia at the lowest concentration (100 ng/cm^2^), where the larval weight was at least 30% higher than in control, indicating that these treatments exerted growth-stimulating effect.

### Histopathological effects of insecticidal proteins on larval midgut

**Fig 2:**
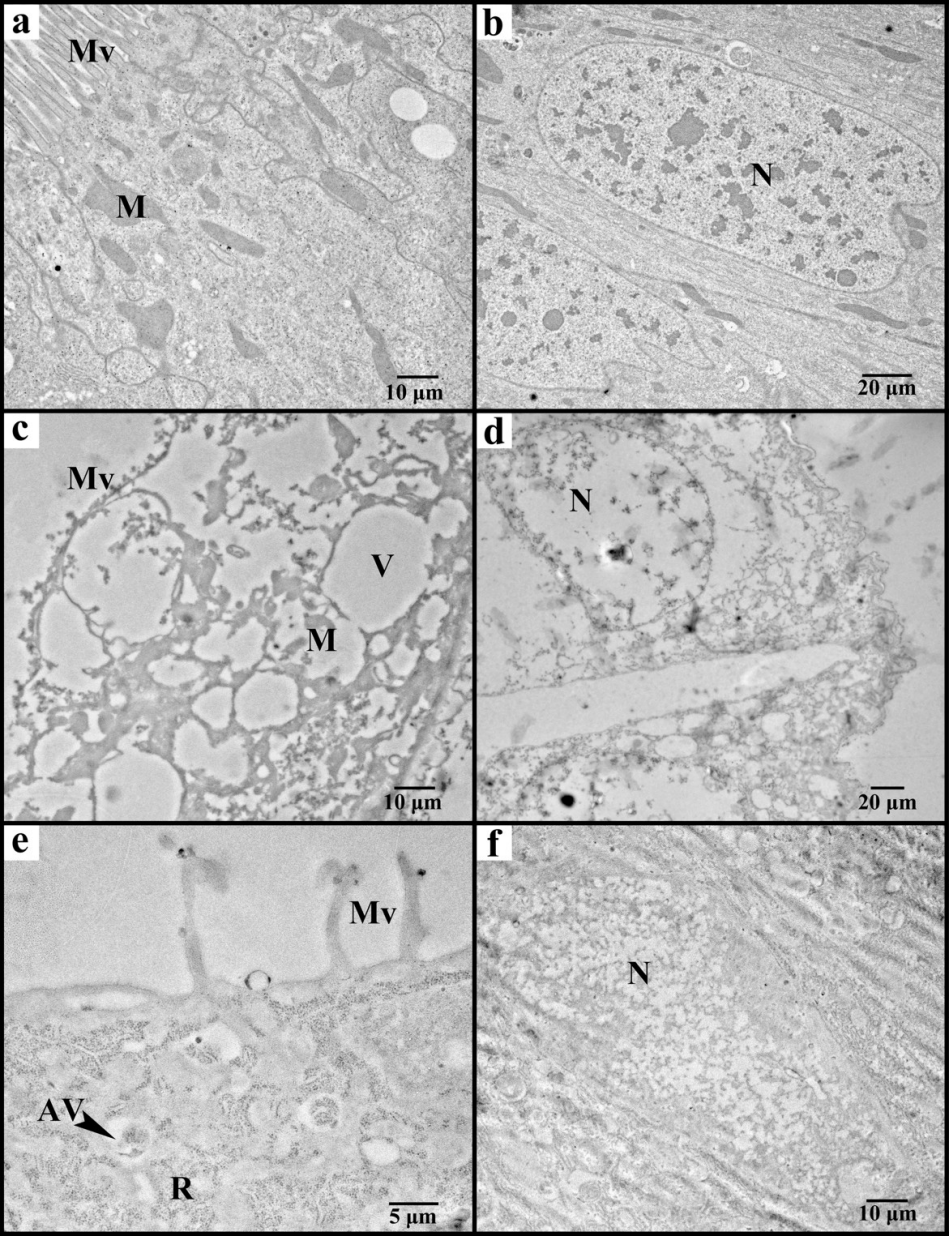
Histopathological effects of *Bacillus thuringiensis* insecticidal proteins on *Agrotis exclamationis* larval midgut. The effects, visible under transmission electron microscope, were captured after 24 h (±4) exposition of larvae to: (a, b) untreated control; (c, d) 1000 ng/cm^2^ of Cry9Ea protoxin; (e, f) 1000 ng/cm^2^ of Vip3Aa protoxin. Av–autophagic vacuole; M–mitochondrion; Mv–microvilli; N–nucleus; R–ribosomes; V–vacuole.

To determine the histopathological effects of Cry9Ea/Vip3Aa proteins on *A*. *exclamationis*, the midguts were isolated after oral ingestion of these toxins by test larvae, and analyzed under transmission electron microscope. The histopathological examination showed that midgut columnar epithelial cells are severely damaged after exposition to insecticidal protein Cry9Ea (Fig 2C and 2D) and Vip3Aa (Fig 2E and 2F), compared to untreated control (Fig 2A and 2B). Analysis of structural changes caused by insecticidal proteins, reveals that the cells have irregularly structured brush border, the microvilli are very short and sparce, and the secretory vesicles disappear at the apical region of the cells. Mitochondria are clearly vacuolated. Likewise, most of the organelles emerge with numerous vacuoles and are destroyed. Intense abnormalities are visible in the nucleus and nuclear sheath. The chromatin is visibly clumped.

## Discussion

*A*. *exclamationis* is a lepidopteran pest species, abundant in Eurasia and northern Africa, causing substantial damage to many crops and garden plants. The susceptibility of *A*. *exclamationis* to safe biocontrol measures such as *Bt* spore-crystal mixtures, nor individual *Bt* Cry/Vip insecticidal proteins has not been determined so far.

In this work the insecticidal potential of *Bt* has been comprehensively assessed towards *A*. *exclamationis*. Primarily, the insecticidal activity of two *Bt* isolates producing different sets of insecticidal proteins has been determined, namely *Bt* subspecies *kurstaki* strain HD-1 and *Bt* subspecies *thuringiensis* strain HD-2. Both microorganisms (or similar strains, representing the same subspecies) are the active ingredients of many commercially available microbial formulations used for biocontrol of lepidopterans [31] and therefore are good models for assessing the efficacy of currently existing sprayable Bt-bioinsecticides against *A*. *exclamationis*. Our results show that both tested strains display rather limited potency against *A*. *exclamationis*. The crystal-spore mixtures derived from these isolates caused less than 50% mortality, even when administered at highest concentration of 850 000 ng/cm^2^. It should be noted that the crystal-spore mixture concentration of 850 000 ng/cm^2^ is much higher than the likely exposure levels received by *A*. *exclamationis* larvae in the area sprayed with commercial insecticides. For example, Dipel® DF (containing 54% of spore-crystal mixture of *Bt* subspecies *kurstaki*) should be applied at maximum concentration of 2 pounds (lb) per acre (https://www.valentbiosciences.com). This gives only ~22 420 ng/cm^2^ of whole formulation available for a pest (assuming the entire surface is just one layer of plant material available to insects). After calculation of spores and crystals contained in the formulation (54%), their final maximum concentration is 12 107 ng/cm^2^. Therefore, the highest concentration of HD-1 and HD-2 spore-crystal mixtures tested in this study is more than 70 times higher than possible concentration of commercial microorganisms in the environment, after application of maximum recommended dosage. Thus, we can speculate that commercially available microbial formulations, which often contain *Bt* isolates from subspecies *kurstaki* or *thuringiensis*, will not be efficient in proper control of *A*. *exclamationis*. Effect caused by application of these bioinsecticides will be insufficient and the surviving larvae may still cause significant and costly losses. Another, more serious possibility is the fast emergence of resistant insect populations caused by sublethal effect exerted by the insecticides.

To explore the *A*. *exclamationis* susceptibility to *Bt* and shed light on low activity of HD-1 and HD-2 strains, the toxicity of six individual *Bt* Cry and Vip proteins has been determined towards this lepidopteran pest. Our results show that only Vip3Aa and Cry9Ea exert significant insect mortality, with the Vip protoxin having almost seven times lower LC_50_ than the Cry. The remaining proteins do not cause more than 50% mortality even when administered to the tested insects at the highest concentration. Similar results could be observed when larvae development was analyzed–Vip3Aa and Cry9Ea protoxins are the most effective in the reduction of larval weight. However, this work shows that also Cry1Aa, Cry1Ia and Cry2Ab induce significant *A*. *exclamationis* growth impairment, especially at high concentrations.

Interesting observation has been made while analyzing results regarding *A*. *exclamationis* larvae development after treatment with toxins Cry1Ca and Cry1Ia. Two higher concentrations of these proteins caused certain degree of larvae growth impairment (or at least did not significantly affect larval growth). However, the lowest concentration of these proteins considerably stimulated the growth of test insects, resulting in ~35–40% higher mean body mass comparing to control treatment. The observed phenomenon is most probably hormesis–“a biphasic dose–response phenomenon characterized by a low-dose stimulation and a high-dose inhibition” [44]. Hormesis most certainly has a negative impact on the field efficacy of a certain Bt-based bioinsecticide, because sublethal doses of the agent can not only be insufficient to stop the pest from damaging crops, but possibly also stimulate his growth and reproductivity. Therefore, Cry1Ca and Cry1Ia are not recommended for the control of *A*. *exclamationis* and probably other closely-related species. Hormesis is widely described as a response to factors such as chemicals, heat, nutrient deprivation, etc. [45]. However, so far, this phenomenon has not been analyzed in research studies elucidating activity of *Bt* insecticidal proteins. Certainly, a deeper investigation of this aspect in the existing literature data and future studies would be helpful to broadly assess hormesis influence on efficiency of existing and newly-adopted Bt-based bioinsecticides.

Since Cry9Ea and Vip3Aa were the only toxins that exerted significant mortality to *A*. *exclamationis* larvae, the histopathological effects of these insecticidal proteins to the insect midgut have been examined. The visible damage of the epithelial cells strongly suggest that larval midgut is the target of both proteins. The histopathological effects of Vip3Aa toxins on larvae other than *A*. *exclamationis* have been shown previously [46–48], but our work is the first report on changes to the insect intestine caused by Cry9E-type protein. The histopathological examinations presented in the above-mentioned articles focused on general disruption of the midgut epithelium (*i*.*e*., swelling/destruction of the columnar cells, loss of the microvilli, stem cell disruption). Our observations were made on TEM micrographs taken with higher magnifications. As a result, in addition to cell destruction and microvilli degeneration also ultrastructural abnormalities of the enterocytes could be clearly observed including disruption of nucleus, nuclear sheath and chromatin as well as vacuolization of organelles such as mitochondria. As shown in this work, the observed severe changes were comparable to larvae fed either with Cry9Ea or Vip3Aa, which supports the opinion that despite differences in insect midgut receptors, the general mode of action of Cry and Vip toxins is similar [49]. On the other hand, there is an assumption, stating that “the onset of symptoms develops after 48–72 h after Vip3A toxin ingestion, while it develops within 24 h after ingestion of Cry toxins” [49]. Our observation showing similar changes between Cry- and Vip-fed *A*. *exclamationis* larvae only 24 h after toxin administration puts into question, if the above-mentioned statement is universal. Perhaps it depends on tested insect, type of toxin or other factors and therefore it requires separate investigation to elucidate this curious matter, which was not the goal of this work.

The toxicity of Cry/Vip proteins has been estimated towards *A*. *exclamationis* for the first time and therefore there is no available comparative data in the literature. However, other *Agrotis* species have been tested for their vulnerability to *Bt* insecticidal proteins. For instance, *A*. *ipsilon* (black cutworm) has low susceptibility (or none at all) to most of the tested Cry1-type proteins [50], with the exception of Cry1Aa and Cry1Fb that exert some level of activity [50]. Interestingly, Cry9Ca is much more active against *A*. *ipsilon* than Cry1 toxins [50]. Other bioassays showed also that Cry9Ca is effective against *A*. *segetum* [51] and Cry9Cb is toxic to *A*. *ipsilon* [52]. Finally, Vip3Aa protein shows significant activity against both *A*. *ipsilon* [53, 54] and *A*. *segetum* [55, 56]. The activity of Cry9E-type proteins has not been tested previously, against any *Agrotis* larvae. High potency of Bt toxins of groups Cry9 and Vip3 should be taken into consideration in future biocontrol strategies against cutworms.

The results of bioassays obtained for single, heterologously-expressed Cry/Vip proteins perfectly explain low activity of two *Bt* strains: HD-1 and HD-2. These bacteria produce mainly Cry1-type and Cry2-type insecticidal proteins, which were shown to exert very limited potency against *A*. *exclamationis*. Thus, commercially-available *Bt* formulations based on *kurstaki* or *thuringiensis* subspecies (or similar subspecies synthetizing mostly Cry1/Cry2 proteins) cannot be used for successful control of heart-and-dart moth. Moreover, we can speculate that also most of the *Bt*-crops registered and/or planted around the world will not be protected against this pest, because the existing and newly adopted cultivars (including *A*. *exclamationis* host plants such as maize) produce mainly Cry1-type or Cry2-type proteins in their tissues [57, 58]. Judging on the results obtained in this work, bioinsecticides consisting of Vip3Aa and Cry9Ea (either in the form of microbial formulations or *Bt*-crops) should be used to effectively manage *A*. *exclamationis* and possibly other closely-related pests.

Noteworthy is the fact that *Bt* Vip3Aa toxin exerts high potency against *A*. *exclamationis*–an organism that has low (or negligible) susceptibility to Cry proteins, as shown in this work. Similar patterns can be observed when analyzing literature data, where insects with low susceptibility to certain Cry1-type toxins (*A*. *ipsilon*, *C*. *pomonella*, *S*. *frugiperda*, *S*. *exigua*) are highly affected by Vip3Aa [27, 40, 42, 59]. Therefore, Vip proteins seem to have much broader species-specificity than Cry proteins. This should be strongly considered when planning pest control strategies using this group of *Bt* toxins. If the broad target spectrum of Vip3A proteins goes together with their high activity, it may lead to a significant augment of biocontrol attempts against economically-important lepidopteran pests. Additional advantage of Vip toxins is the lack of any apparent sequence similarity to Cry proteins, proven affinity to different receptors in insect midgut and low probability of significant cross-resistance in insect populations, which was broadly reviewed in recent publications [35, 49, 60, 61]. This makes Vip proteins perfect candidates to augment the existing biocontrol strategies by: (i) broadening target range of *Bt*-based bioinsecticides; (ii) providing higher mortality in lepidopteran pests; and (iii) controlling the emerging Cry-resistant insect populations. On the other hand, broad target range of Vip proteins might be a sign of possible cross-order or cross phylum activity. Up to now, adverse effects of Vip3Aa towards nontarget organisms (NTOs), such as bees, earthworms, ladybirds, mice, etc., have been ruled out. The only exception was slight growth impairment of *Daphnia magna* exposed to Vip3Aa20, although the tested observed adverse effect concentration (NOAECs) of the protein was much higher than the estimated environmental concentrations (EECs) of the insecticidal protein in the field [62]. On the other hand, the number of NTOs tested so far is rather limited and therefore further approaches should be undertaken to fully assess the off-target activity of Vip3-type proteins and comprehensively confirm their environmental safety.

Cry9Ea studied in this work is a member of Cry9 group of *Bt* toxins, currently comprising of 15 known proteins (Cry9Aa, Ba, Bb, Ca, Cb, Da, Db, Dc, Ea, Eb, Ec, Ed, Ee, Fa, Ga) as summarized in BPPRC database [37]. Cry9 proteins are considered lepidopteran-active part of the *Bt* arsenal, similarly to Cry1, Cry2 or Vip3 toxins, however they are much less studied than the latter. A few reports suggest that Cry9 proteins are recognized by different receptors in insect midgut comparing with Cry1/Cry2 –therefore, low probability of insect cross-resistance to these diverse groups of toxins exists [63, 64]. Much still remains unknown however, regarding target species range of Cry9 insecticidal proteins. Until 2009 only four Cry9-type toxins (Cry9Aa, Cry9Bb, Cry9Ca, Cry9Ec) were bioassayed against very limited number of pest species [28]. This work extends the existing knowledge, regarding species specificity of members of Cry9 group, showing significant activity of Cry9Ea towards *A*. *exclamationis*. Moreover, recent study has demonstrated that Cry9Ea is one of the most potent *Bt* proteins against *C*. *pomonella–*an abundant pest of apple orchards, representing the Tortricidae family [42]. It is probable, that high bioinsecticidal potential is yet hidden among toxins of *Bt* Cry9 group.

In conclusion, *Bt* Cry9Ea and Vip3Aa toxins show significant activity against *A*. *exclamationis* cutworm larvae–pest species with low or negligible sensitivity to Cry1/Cry2 proteins and thus not susceptible to most of the currently used *Bt*-based bioinsecticides. Apart from their high activity, Cry9Ea and Vip3Aa toxins share little or no homology with “overused” Cry1/Cry2 toxins, they are recognized by different receptors in insect midgut [56, 63–66], and therefore display low risk of cross-resistance. The results of this work and other literature data suggest that Cry9Ea and Vip3Aa (and possibly other Cry9/Vip3 proteins) display high implementation potential in developing new biocontrol strategies, which may augment the existing approaches and sustain the use of *Bt*-based bioinsecticides for the next decades.

## Supporting information

S1 File Detailed experimental procedures. (DOCX)

S2 File Results showing obtained microbial formulations and insecticidal proteins. (DOCX)

S3 File Activity of microbial formulations and insecticidal proteins towards susceptible target species (positive controls). (DOCX)

S1 Raw images (PDF)
