# Correction: Insecticidal activity of *Bacillus thuringiensis* towards *Agrotis exclamationis* larvae–A widespread and underestimated pest of the Palearctic zone

**DOI:** 10.1371/journal.pone.0332032

**Published:** 2025-09-10

**Authors:** The PLOS One Staff

In the Abstract section, there is an error in the first and third sentences. The correct first sentence is: *Bacillus thuringiensis* is an entomopathogenic bacterium commonly used as a bioinsecticide against numerous invertebrate pests. The correct third sentence is: To investigate this matter, the biological activity of six selected (Cry1Aa, Cry1Ca, Cry1Ia, Cry2Ab, Cry9Ea and Vip3Aa), heterogously-expressed *B. thuringiensis* insecticidal proteins has been assessed towards *A*. *exclamationis*.

The publisher apologizes for the errors.

## References

[1] Baranek J, Jakubowska M, Gabała E. Insecticidal activity of *Bacillus thuringiensis* towards *Agrotis exclamationis* larvae–A widespread and underestimated pest of the Palearctic zone. PLoS ONE. 2023;18(3):e0283077. doi: 10.1371/journal.pone.0283077 36928078 PMC10019718

