# Supplementary material for: Insecticidal activity of Bacillus thuringiensis towards Agrotis exclamationis larvae–A widespread and underestimated pest of the Palearctic zone
Source: PLoS One. 2023 Mar 16;18(3):e0283077. doi: 10.1371/journal.pone.0283077 (PMC10019718; doi:10.1371/journal.pone.0283077)
Supplement: S1 File — (DOCX) [file pone.0283077.s001.docx]

**S1 File. Detailed experimental procedures.**

**Insecticidal activity of *Bacillus thuringiensis* towards *Agrotis exclamationis* larvae – a widespread and underestimated pest of Palearctic zone**

Jakub Baranek^1^*, Magdalena Jakubowska^2^, Elżbieta Gabała^3^

^1^Department of Microbiology, Faculty of Biology, Adam Mickiewicz University in Poznań, Uniwersytetu Poznańskiego 6, 61-614, Poznań, Poland

^2^Department of Monitoring and Signalling of Agrophages, Institute of Plant Protection-National Research Institute, Władysława Węgorka 20, 60-318 Poznań, Poland

^3^Institute of Plant Protection-National Research Institute, Władysława Węgorka 20, 60-318 Poznań, Poland

*Corresponding author: jakbar@amu.edu.pl [JB]

**Detailed experimental procedures**

1. **Preparation and quality control of microbial formulations**

Bacterial strains were routinely cultured for 24 h at 30°C on Brain Heart Infusion (BHI) medium (Oxoid), supplemented with 1.5% agar (BioCorp). To obtain endospores and protein crystals the bacterial isolates were cultured for 4-5 days at 30°C on the on Sporulation Medium (SM) (0.75% peptone; 50 mM KH_2_PO_4_; 1 mM CaCl_2_ x 2H_2_O; 0.5 mM MgSO_4_ x 7H_2_O; 0.05 mM ZnSO_4_ x 7H_2_O; 0.05 mM Fe_2_(SO_4_)_3_; 0.01 mM MnSO_4_ x H_2_O; 0.3 mM H_2_SO_4_; 1.5% agar; pH 7.2), which is a slightly modified BP medium described before [1]. After collection, the spore-crystal mixture was washed three times with MilliQ water, weighted and kept as water suspension at -70°C until use. The sporulation level and parasporal crystal formation were checked by staining the endospore-crystal smear on glass slide, using method described earlier [2] with slight modifications, as follows. The microbiological material was suspended in demineralized water and spread as thin layer on degreased glass slide, allowed to dry and heat-fixed. The smear was covered with amido black solution (1.5% amido black 10 B (w/v), 50% methanol (v/v); 10% (v/v) acetic acid) for 50 seconds. Next, the stain was removed by rinsing the slide with demineralized water. Subsequently, the smear was covered with Ziehl’s carbol fuchsin (1% fuchsin (w/v), 5% phenol (w/v), 10% ethanol (v/v)) for 20 seconds and rinsed with demineralized water. Next, the slide was gently wiped with blotting paper and air-dried. Stains were then examined under light microscope (Olympus CX21LED) at 1000× magnification. Both staining solutions used in the above procedure were filtered through 0.45 µm syringe filters before use.

The spore-crystal mixtures were also examined via scanning electron microscope. To achieve this, the spore-crystal mixtures kept at -70°C were thawed, spread on plexiglass surface and allowed to dry. Subsequently the samples were silver-coated and checked using Zeiss Evo 40 scanning electron microscope.

1. **Insecticidal protein (Cry/Vip) expression and extraction**

The expression vectors were transformed into chemically competent *E. coli* BL21 (DE3) codon plus cells, using calcium chloride method. The transformants were incubated in LB medium supplemented with ampicillin (concentration 100 µg/ml) until OD_600_ reached 0.8-1. Next, the isopropyl β-d-1-thiogalactopyranoside (IPTG; Thermo Scientific) was added to a final concentration of 1 mM and the cultures were further incubated for 18-24 h at room temperature. The cells were pelleted in centrifuge (10 000 × g, 10 min, 4°C) and frozen in -20°C. Next, the cells were lysed in ice-cold lysis buffer (50 mM Tris/HCl, pH 8; 100 mM NaCl; 1 mM EDTA) and supplemented with phenylmethylsulfonyl fluoride (PMSF; BioShop) and lysozyme (Sigma Aldrich) to a final concentration of 1 mM and 0.25 mg/ml, respectively. One gram of cell debris was typically suspended in 5-8 ml of the lysis buffer and kept for 30 min at room temperature, with shaking (50 rpm). Subsequently, deoxycholic acid (MP Biomedicals) was added to a final concentration of 0.8-1.3 mg/ml and cell suspensions were cooled on ice and sonicated with several pulses (22 kHz frequency, 14 mm amplitude), ten seconds each, using ultrasound disintegrator (UD-11; Techpan). Lysates containing Cry protoxins expressed as insoluble fractions (namely Cry1Aa, Cry1Ca, Cry2Aa, Cry9Ea) were centrifuged 40 000 × g, 4°C, and the Cry-protein-rich pellets were washed twice in 12 ml of washing buffer (50 mM Tris/HCl, pH 8; 100 mM NaCl; 10 mM EDTA; 0.5% (v/v) Triton X-100) and resuspended in 5 ml of solubilization buffer (50 mM Na_2_CO_3_, pH 10; 10 mM DTT) per gram of original cell pellet. After 18-24 h of incubation in 4°C, the lysates were cleared by centrifugation 40 000 × g, 10 min, 4°C and the supernatants containing dissolved Cry proteins were collected. Lysates containing Cry protoxins produced mainly as soluble fractions (namely Cry1Ia, Vip3Aa) were only centrifuged 40 000 × g, 10 min, 4°C and the Cry/Vip-protein-rich supernatant was collected. All samples containing the insecticidal proteins were subsequently passed through 0.22 µm syringe filters (CarlRoth) and dialyzed 48 h in 14 kDa MWCO Visking tubes (Carl Roth) with 4-5 exchange of the dialysis buffer (50 mM Tris/HCl, 100 mM NaCl, pH 9). Simultaneously, as a control, the non-transformed *E. coli* BL21 (DE3) codon plus was processed using the same protocols as described above, only without antibiotic selection during culturing steps. All the obtained preparations, containing pesticidal proteins, as well as the control were aliquoted and kept at -70°C until use.

The protein content was verified using 10% sodium dodecyl sulfate polyacrylamide gel electrophoresis and Cry/Vip toxin concentrations were estimated by densitometry with known amounts of bovine serum albumin as standards, using GelAnalyzer 2010a software (AnalystSoft Inc.). Theoretical masses of the expressed Cry/Vip proteins were calculated upon corresponding deduced amino acid sequences using Protein Molecular Weight Calculator online tool (Science Gateway; <https://www.sciencegateway.org/tools/proteinmw.htm>).

REFERENCES

1. Lecadet MM, Blondel MO, Ribier J. Generalized transduction in *Bacillus thuringiensis* var. *berliner* 1715 using bacteriophage CP-54Ber. J Gen Microbiol. 1980;121: 203–212. doi:10.1099/00221287-121-1-203

2. Smirnoff WAJ. A staining method for differentiating spores, crystals, and cells of *Bacillus thuringiensis* (Berliner). Journal of Insect Pathology. 1962;4: 384–386.
