# Supplementary material for: Insecticidal activity of Bacillus thuringiensis towards Agrotis exclamationis larvae–A widespread and underestimated pest of the Palearctic zone
Source: PLoS One. 2023 Mar 16;18(3):e0283077. doi: 10.1371/journal.pone.0283077 (PMC10019718; doi:10.1371/journal.pone.0283077)
Supplement: S2 File — (DOCX) [file pone.0283077.s002.docx]

**S2 File. Results showing obtained microbial formulations and insecticidal proteins**

**Insecticidal activity of *Bacillus thuringiensis* towards *Agrotis exclamationis* larvae – a widespread and underestimated pest of Palearctic zone**

Jakub Baranek^1^*, Magdalena Jakubowska^2^, Elżbieta Gabała^3^

^1^Department of Microbiology, Faculty of Biology, Adam Mickiewicz University in Poznań, Uniwersytetu Poznańskiego 6, 61-614, Poznań, Poland

^2^Department of Monitoring and Signalling of Agrophages, Institute of Plant Protection-National Research Institute, Władysława Węgorka 20, 60-318 Poznań, Poland

^3^Institute of Plant Protection-National Research Institute, Władysława Węgorka 20, 60-318 Poznań, Poland

*Corresponding author: jakbar@amu.edu.pl [JB]

**Results showing obtained microbial formulations and insecticidal proteins**

1. **Microbial formulations**

Microbial formulations containing endospores and parasporal crystals of *Bt* subsp. *kurstaki* HD-1 and *Bt* subsp. *thuringiensis* HD-2 were obtained via vegetative cell sporulation. Microscopic observation confirmed production of endospores and parasporal crystal bodies in both isolates (S1 and S2 Figs). Strain HD-1 forms two types of crystals – bipyramidal and cuboidal (S1a and S2a Figs), which is in line with previous report regarding this strain [1]. Strain HD-2 produces bipyramidal crystals (S1b and S2b Figs).


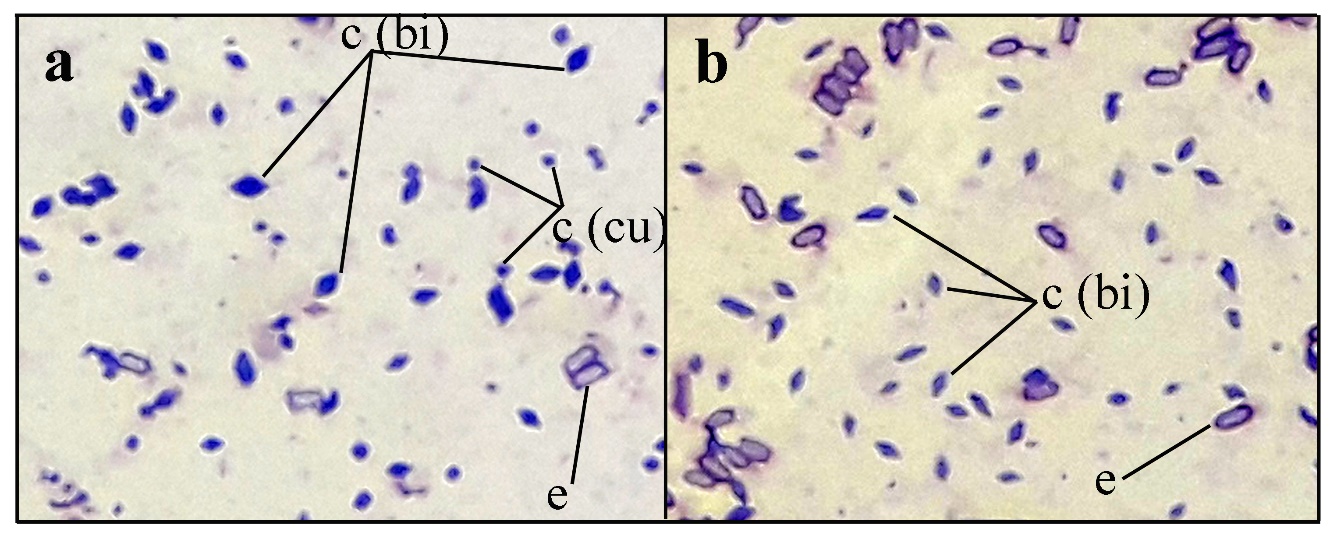


**S1 Fig.** *Bacillus thuringiensis* subspecies *kurstaki* HD-1 (a) and *B. thuringiensis* subsp. *thuringiensis* HD-2 (b) post sporulation, under light microscope (magnification 1000×). Objects visible on the images include endospores (e) as well as parasporal crystals (c) with bipyramidal (bi) or cuboidal (cu) shape

**
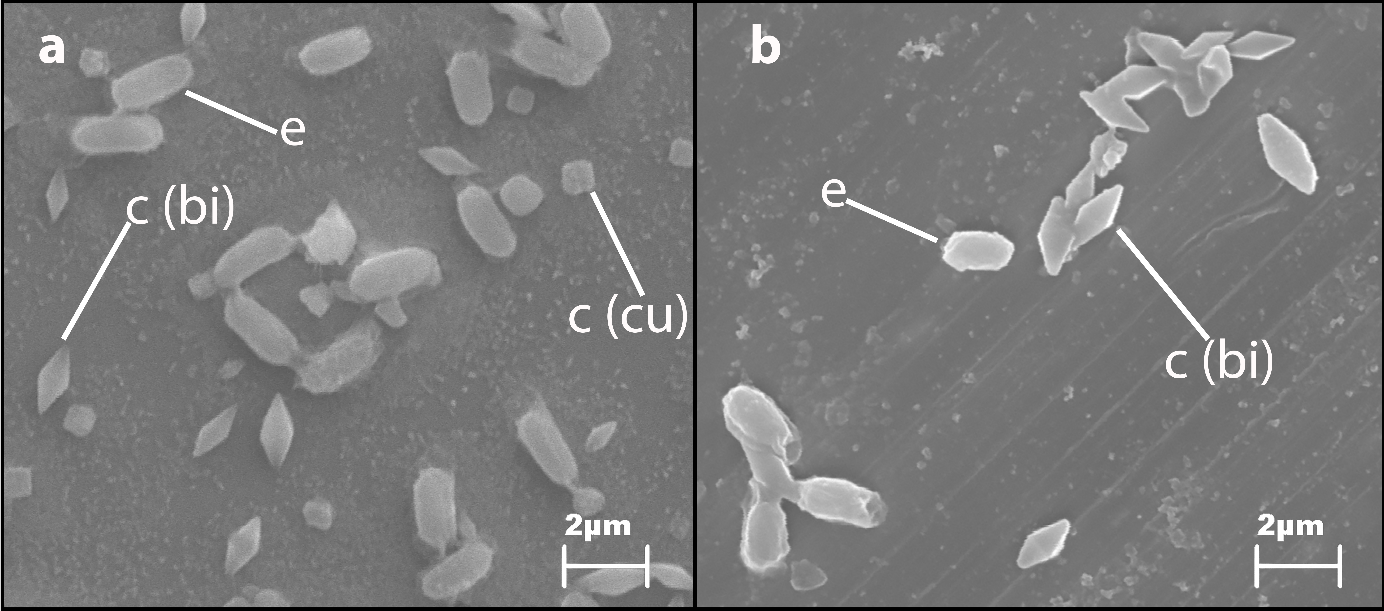
S2 Fig.** *Bacillus thuringiensis* subspecies *kurstaki* HD-1 (a) and *B. thuringiensis* subsp. *thuringiensis* HD-2 (b) post sporulation, under scanning electron microscope. Objects visible on the images include endospores (e) as well as parasporal crystals (c) with bipyramidal (bi) or cuboidal (cu) shape.

1. **Heterologously-expressed Cry/Vip proteins**

Expression vectors harboring genes encoding six *Bt* proteins were transformed into *E. coli* BL21 (DE3) codon+ cells and heterologous expression was carried out. Bacterial proteins were extracted from cell lysates and resolved on SDS-PAGE. The desired Cry and Vip proteins were distinguished via electrophoresis as apparent bands with expected molecular weights: Cry1Aa (~133 kDa), Cry1Ca (~135 kDa), Cry1Ia (~81 kDa), Cry2Ab (~70 kDa), Cry9Ea (~130 kDa), Vip3Aa (~88 kDa), and they were not present in the control (S3 Fig).





**S3 Fig.** SDS-PAGE analysis of *Bacillus thuringiensis* protoxins present in transformed *Escherichia coli* BL21 (DE3) cell lysates after expression and protein extraction. Line numbers indicate lysates containing proteins: 1 – Cry1Aa; 2 – Cry1Ca; 3 –Cry1Ia; 4 – Cry2Ab; 5 – Vip3Aa; 6 – Cry9Ea. Arrowheads indicate bands corresponding with the Cry/Vip proteins. C – control; M – protein weight standard (Thermo Scientific, Cat. no. 26614). The lysate containing Cry9Ea protein was resolved on different SDS-PAGE gel than the remaining samples, which is denoted by vertical black line visible on the figure. Both original, uncropped gel images are provided in Supporting information.

**REFERENCE**

1. Nair K, Al-Thani R, Al-Thani D, Al-Yafei F, Ahmed T, Jaoua S. Diversity of *Bacillus thuringiensis* strains from Qatar as shown by crystal morphology, δ-Endotoxins and Cry gene content. Frontiers in Microbiology. 2018;9: 1–10. doi:10.3389/fmicb.2018.00708
