# Supplementary material for: Insecticidal activity of Bacillus thuringiensis towards Agrotis exclamationis larvae–A widespread and underestimated pest of the Palearctic zone
Source: PLoS One. 2023 Mar 16;18(3):e0283077. doi: 10.1371/journal.pone.0283077 (PMC10019718; doi:10.1371/journal.pone.0283077)
Supplement: S3 File — (DOCX) [file pone.0283077.s003.docx]

**S3 File. Activity of microbial formulations and insecticidal proteins towards susceptible target species (positive controls).**

**Insecticidal activity of *Bacillus thuringiensis* towards *Agrotis exclamationis* larvae – a widespread and underestimated pest of Palearctic zone**

Jakub Baranek^1^*, Magdalena Jakubowska^2^, Elżbieta Gabała^3^

^1^Department of Microbiology, Faculty of Biology, Adam Mickiewicz University in Poznań, Uniwersytetu Poznańskiego 6, 61-614, Poznań, Poland

^2^Department of Monitoring and Signalling of Agrophages, Institute of Plant Protection-National Research Institute, Władysława Węgorka 20, 60-318 Poznań, Poland

^3^Institute of Plant Protection-National Research Institute, Władysława Węgorka 20, 60-318 Poznań, Poland

*Corresponding author: jakbar@amu.edu.pl [JB]

**Activity of microbial formulations and insecticidal proteins towards susceptible target species (positive controls).**

1. **Insect bioassays**

To verify the biological activity of all *B. thuringiensis* strains and insecticidal proteins used in this study, they were bioassayed against susceptible insects – either *Cydia pomonella* (in case of Cry1Aa, Cry1Ia, Cry2Ab and Cry9Ea toxins) or *Spodoptera exigua* (in case of Cry1Ca, and Vip3Aa toxins as well as spore-crystal mixtures derived from HD-1 and HD-2 strains). All tested strains/toxins has proven their activity by causing high (>75%) mortality in insect larvae (**S1** **Table**).

**S1** **Table.** Mortality of *Cydia pomonella* ad *Spodoptera exigua* larvae after treatment with *B. thuringiensis* (*Bt*) spore-crystal mixtures and heterogously-expressed individual insecticidal toxins.

| Strain/Toxin | Concentration (ng/cm^2^) | Mortality (%)+SE^a^ | |
| --- | --- | --- | --- |
|  |  | *Cydia pomonella*^b^ | *Spodoptera exigua*^c^ |
| HD-1 | 50 000 | NT^d^ | 77.1 (±22.9) |
| HD-2 |  | NT | 95.8 (±4) |
| Cry1Aa | 1000 | 100 (±0.0) | NT |
| Cry1Ca |  | NT | 83.3 (±7.2) |
| Cry1Ia |  | 91.6 (±8.3) | NT |
| Cry2Ab |  | 100.0 (±0.0) | NT |
| Cry9Ea |  | 100.0 (±0.0) | NT |
| Vip3Aa |  | NT | 76.4 (±13.6) |

^a^ Mean mortality and standard error of the mean (SE) of three (for *Bt* toxins) or two (for *Bt* strains) independent replications, scored seven (for *S. exigua*) or ten (for *C. pomonella*) days after treatment

^b^ First instar (1-2 day-old) *C. pomonella* larvae

^c^ First instar (2-3 day-old) *S. exigua* larvae

^d^NT – not tested
