# Supplementary material for: Insecticidal activity of Bacillus thuringiensis towards Agrotis exclamationis larvae–A widespread and underestimated pest of the Palearctic zone
Source: PLoS One. 2023 Mar 16;18(3):e0283077. doi: 10.1371/journal.pone.0283077 (PMC10019718; doi:10.1371/journal.pone.0283077)
Supplement: S1 Raw images — (PDF) [file pone.0283077.s004.pdf]

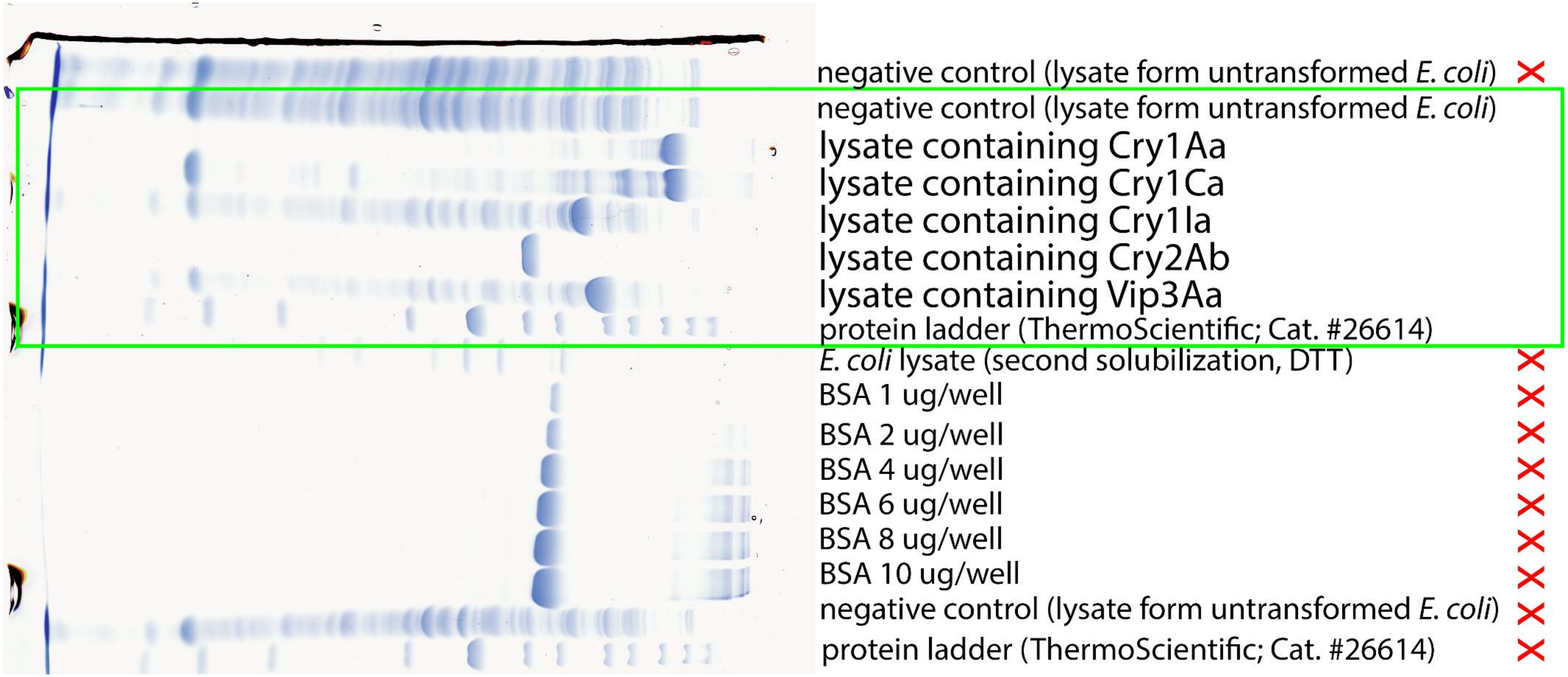

The SDS-PAGE gel was captured using Epson V700 scanner. The lanes marked with green rectangle were included in S3 Fig presented in S2 File.

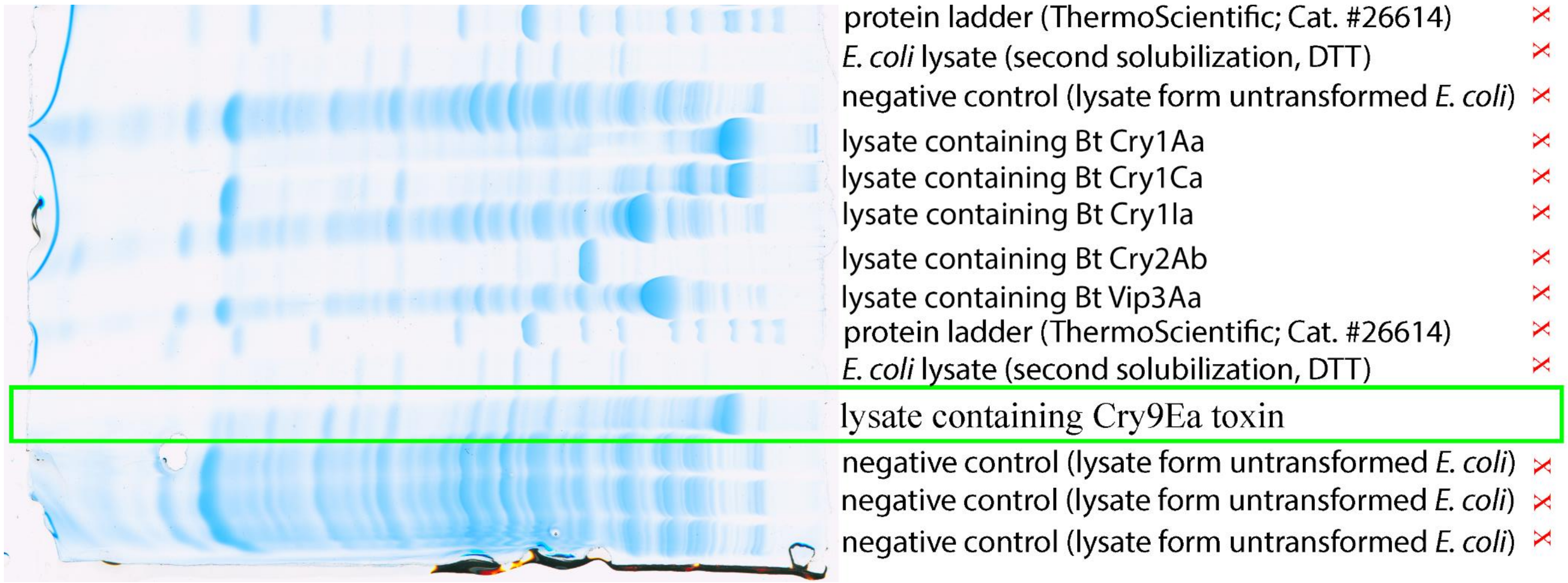

The SDS-PAGE gel was captured using Epson V700 scanner. The lane marked with green rectangle was included in S3 Fig presented in S2 File.
